# The Present Number of "Journal"

**Published:** 1877-11

**Authors:** 


This Number is a good one, is it not? We feel that the several articles, original and selected, are quite up to the average of similar productions in other professions, and are no mean evidence of the industry and talent, as well as the cultivation of the Dental Profession.

The article (reprinted from the *Dental Register*,) on the "Action of the Nervous System," by J. J. Caldwell, of this city, is worthy of careful perusal. Dr. C. is a gentleman eminently well qualified to write on this subject, having made it a special study for years, and is withal a man of fine acquirements added to his natural gifts, with a good many years hospital practice, and a writer well known to the medical public. Other articles are promised from Dr. Caldwell, and we hope to see the pages of the Journal well crowded.

